# Experience of Conjugality Among Older People in Long-Term Care Facilities: A Scoping Review

**DOI:** 10.1177/07334648251369264

**Published:** 2025-08-26

**Authors:** Florbela Bia, Zaida Charepe, Cristina Marques-Vieira

**Affiliations:** 1 Center for Interdisciplinary Research in Health (CIIS), 59207 Universidade Católica Portuguesa, Lisbon, Portugal; 2 School of Nursing São João de Deus, Nursing Department, University of Évora, Évora, Portugal; 3 Faculty of Health Sciences and Nursing, Universidade Católica Portuguesa, Lisbon, Portugal; 4 86712 Nursing School of Lisbon (ESEL), Lisbon, Portugal; 5 Innovation and Development Center of Lisbon (CIDNUR), 86712 Nursing School of Lisbon(ESEL), Lisbon, Portugal

**Keywords:** population aging, institutional care, marriage, marital relationships, nursing homes, long-term care facilities

## Abstract

This scoping review examines the underexplored impact of the transition to residential care on conjugal relationships among older couples in long-term care facilities (LTCFs). With an aging population and evolving care policies, understanding marital experiences in residential care settings becomes increasingly essential. Using the Joanna Briggs Institute methodology and the Population, Concept, and Context framework, a systematic search across six databases identified 17 studies from 529 articles. These studies were classified into three living arrangements: spouses in LTCFs with partners in the community, co-residing couples, and those addressing both scenarios. Findings reveal variations in living arrangements and the emotional complexity of these transitions, emphasizing the importance of maintaining marital bonds and relational identity. Influenced by institutional care policies and staff training, these outcomes highlight the need for longitudinal, quantitative and intervention-based research. Policy recommendations advocate relationship-centred care to promote privacy, marital continuity, and interventions enhancing marital well-being in LTCFs.

**What this paper adds**

• Examines how admission to long-term care facilities disrupts marital roles, intimacy, and emotional well-being among older adults.
• Identifies research gaps, including the limited focus on co-residing couples, the predominance of qualitative studies, and the need for structured interventions.

**Applications of study findings**

• Underscores the need for policy reforms that integrate relational well-being into LTCF models to support marital relationships.
• Advocates for cross-national comparisons and the development of evidence-based interventions to enhance relationship-centred care in long-term care (LTC) settings.

## Introduction

Global aging is one of the most pressing social, demographic, and healthcare challenges of the 21st century (World Health Organization [WHO], 2021). Reports from international organizations, including the United Nations [UN], (2021), the Organisation for Economic Co-operation and Development [OECD], (2025), and the European Commission [EC], (2023), show a steady increase in life expectancy, leading to a greater demand for LTC services. The proportion of the population aged 65 and older has reached unprecedented levels, raising complex concerns about the care for older adults, marital well-being, and couples’ quality of life in residential structures. Aging-related health declines often necessitate transitions to LTCFs (Bárrios et al., 2020; Gu et al., 2021). These transitions can disrupt not only individual autonomy but also relational and emotional bonds, particularly marital relationships.

Although most reaserch on aging and LTC focuses on healthcare delivery, functional dependence, and institutional care models, limited attention has been paid to how older couples navigate conjugal relationships within these settings (Glasier & Arbeau, 2019; Soulsby et al., 2022). Marital bonds are a key source of emotional well-being, social support, and identity continuity in later life. However, sustaining these relationships in LTCFs is often tricky. Challenges stem not only from care practices but also from institutional policies and infrastructural limitations that fail to adequately address the relational needs of older couples (Abrahamson et al., 2009; McAuliffe et al., 2023; Torgé, 2020).

According to WHO (2002), building and maintaining relationships is a fundamental need for older people. Marital relationships in later life are, in particular, linked to better quality of life, emotional well-being, and overall health. Conjugal relationships significantly contribute to these consequences. Recent research indicates that spousal emotional support enhances happiness (Abaei & Martin, 2024), while trust and intimacy are key to relationship satisfaction (Batool et al., 2025). Regular contact with close others also promotes planning and reduces stress during life transitions (Barrett et al., 2024; Chacon & Pedroso, 2020).

Despite these benefits, conjugal relationships in later life face some challenges. They require continuous communication and emotional effort (Hähnel, Töpfer, & Wilz, 2023), are shaped by societal perceptions (Lefévre, 2023) and often reflect gender-based differences (Abaei & Martin, 2024). Understanding these dynamics is vital to fostering environments that support conjugality in later life.

Conjugality is widely recognized as a protective measure for older couples (Holton et al., 2023; Liu et al., 2019; Robertson et al., 2023). It plays a crucial role in mitigating loneliness and social isolation. Scorsolini-Comin and dos Santos (2010) define conjugality as a distinctive relational model uniquely constructed by each couple of two individuals. It serves as a space for interaction between the “I” and the “We”, wherein two individuals build a guiding framework for their relational dynamics (Chacon & Pedroso, 2020).

Despite growing global efforts to reform LTC policies, most care models remain individual-centred, often overlooking the dimensions of marital relationships (Shaw & Csikai, 2024). While recent policy frameworks promote person-centred care (EC, 2023; WHO, 2021), practical implementation in LTCFs remains inconsistent. Few institutions provide explicit guidelines for spousal cohabitation, privacy accommodations (Hallam, 2018), or emotional support for separated couples (Olsen et al., 2019). McAuliffe et al. (2023) emphasise the need for clearer institutional policies to support staff in making consistent and informed decisions. Similarly, Lefévre (2023) and Grigorovich et al. (2024) stress the importance of structured training programmes and written policies that recognise and legitimise the intimacy among older adult couples in care settings.

In addition to these perspectives, Culberson et al. (2017) provide a set of explicit and practical guidelines. These include developing comprehensive policy frameworks, continuous professional training, interdisciplinary assessments at admission, and access to private spaces that facilitate intimate relationships. They also recommend routine policy reviews to ensure continued relevance and effectiveness.

Research from Nordic European countries (Rostgaard et al., 2022) and Canada (Kirkham et al., 2024; Lee et al., 2024) suggests that gaps in regulatory frameworks related to relational well-being persist despite progress in LTCF regulation. These gaps necessitate further research to identify knowledge deficits, methodological trends, and political implications concerning marital relationships in LTC settings.

Nurses and other healthcare professionals need to acquire a thorough understanding of conjugal relationships among older adults in institutional care settings. Lefévre (2023) demonstrates that both staff knowledge and institutional policies play a significant role in shaping the acceptance of intimacy within LTFs. Therefore, staff training and the development of supportive institutional guidelines are critical to enhance the well-being and quality of life of cohabiting older couples.

### Research Aims and Scope

This study aims to map the experiences of older couples in LTCFs systematically. It explores how residential care structures shape their conjugal dynamics. The research also identifies existing knowledge gaps and methodological trends regarding conjugal relationships in residential settings.

## Methods

We conducted this scoping review using the Joanna Briggs Institute (JBI) methodology, guided by the framework established by Arksey and O’Malley (2005), with refinements by Peters et al. (2024). We employed the Population, Concept, and Context (PCC) approach to map existing knowledge systematically. This method allowed us to include diverse study designs while ensuring a broad yet structured exploration of conjugal relationships in LTCFs (Munn et al., 2018; Peters et al., 2024). We selected the PCC approach for its ability to provide a comprehensive synthesis of the literature while maintaining methodological rigor. The study protocol was prospectively registered on the Open Science Framework (OSF) under the DOI https://doi.org/10.17605/OSF.IO/PF3RE.

### Identifying the Research Questions

(1) What are the main knowledge gaps in the literature on conjugal relationships among couples in LTCFs?
(2) What are the experiences of conjugal relationships among older individuals and their spouses residing in LTC settings, and how do residential structures shape their conjugal dynamics?

### Identifying Relevant Studies

We implemented a structured three-phase search strategy across six electronic databases: RCAAP, CINAHL Complete, MedicLatina, MEDLINE Complete, Psychology and Behavioural Sciences Collection, PsycArticles, and PubMed. Further details of the search strategy are available in Supplemental File 1. In the first phase, we conducted an exploratory search to identify key terms, such as medical subject headings (MeSH), and index terms relevant to conjugal relationships in long-term care facilities, ensuring alignment with discipline-specific terminology. In the second phase, we performed a comprehensive database search using a combination of MeSH terms and free-text keywords to enhance sensitivity across indexing systems. We applied Boolean operators and truncation techniques to increase precision. Finally, in the third phase, we manually screened the reference list to identify additional relevant studies not captured in the database searches, minimizing omission risk.

### Study Selection

We conducted a study selection using Rayyan^®^, a web-based tool designed to streamline screening in systematic reviews, based on the eligibility criteria (Table 1). Our inclusion and exclusion criteria aligned with the study’s objectives. We considered as eligible studies those focused on couples aged over 60 and various forms of conjugality, while we excluded those involving widowed, divorced, single, or solitary individuals. We also excluded studies on hospitalization or short-term care, limiting the context to LTCFs. In our sources, we included quantitative and qualitative studies in Portuguese, English, and Spanish, without time restrictions. We excluded, however, gray literature, prioritizing empirical research explicitly addressing conjugal relationships in LTCFs.

**Table 1.** Inclusion and exclusion criteria

	Inclusion criteria	Exclusion criteria
Participants	Studies that included residents of long-term care facilities and their spouses over the age of 60 (WHO, 2002). The term spouse will be used to define the members of the couple. The level of cognition or comorbidities of the participants were not considered	
Concept	The concept of conjugality was included in a comprehensive way (Scorsolini-Comin & dos Santos, 2010), considering people in religious, civil or common-law marriage. We also included studies that looked at other forms of relationships, namely homoaffective relationships.	Studies that looked at older people with a marital status of widowhood, divorced, single or living alone were excluded.
Context	Residences for older adults, senior residences, nursing homes, long-term geriatric institutions and assisted living residences without geographical limitation	Studies in the context of hospitalization, Continuous Care Units, short- or medium-term care were not included.
Types of sources	Studies in Portuguese, English and Spanish, of a quantitative and qualitative nature, whether experimental, quasi-experimental and meta-analyses.	Theoretical discussions without empirical data

**Figure 1.**
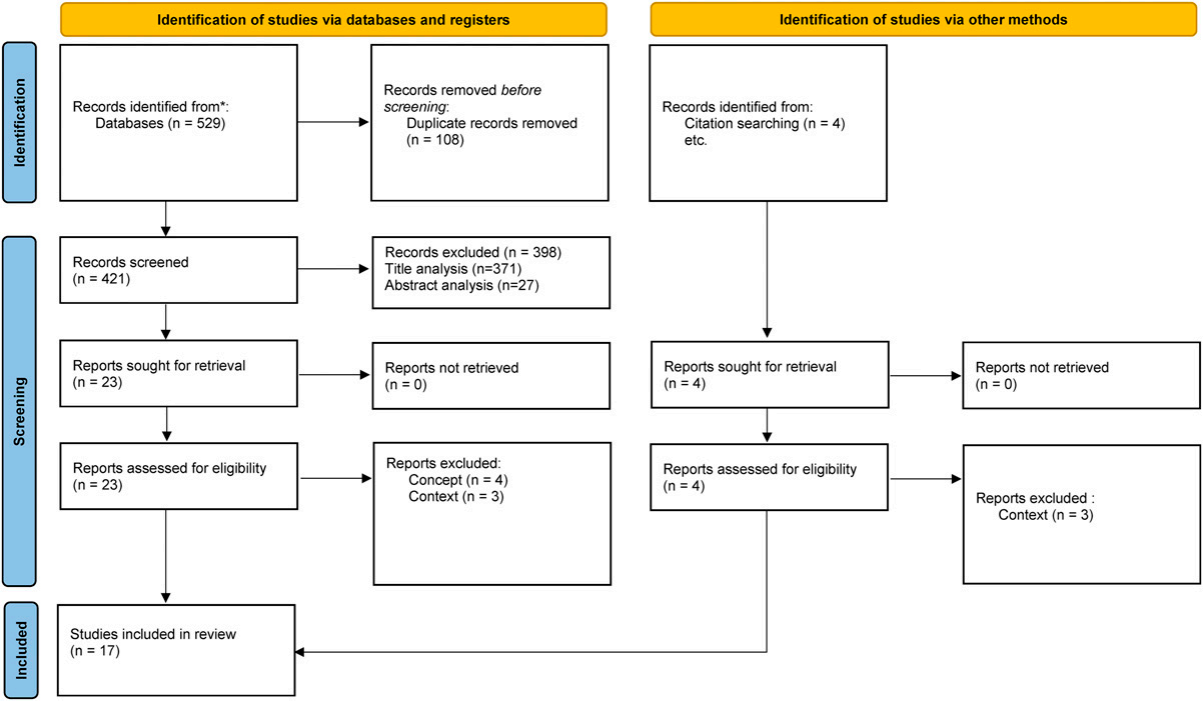
Flow Diagram of Study Selection Process (Page et al., 2021)

Two authors independently conducted the eligibility screening by reviewing the titles, abstracts, and full texts of the articles. They resolved disagreements through discussion or, if needed, third-party arbitration. The flowchart in Figure 1 presents a detailed visualization of the screening process.

For studies that met the eligibility criteria, we used a standardized charting form to extract key methodological and thematic elements. Extracted data included study characteristics (i.e., publication year, geographic location, disciplinary field, and study objectives) along with methodological details (i.e., research design, data collection methods, and sample composition). We then systematically organized information on key findings and thematic categorizations to ensure consistency with the review objectives.

We analysed the extracted data using thematic synthesis to facilitate a structured interpretation of findings while maintaining methodological transparency (Tricco et al., 2018). This analytical process allowed us to identify common themes and divergences across studies, ensuring that relational and environmental factors influencing conjugal experiences in LTCFs were thoroughly examined.

### Data Charting and Reporting the Results

We extracted relevant information to the research questions using the JBI guidelines (Peters et al., 2024). Table 2 provides a summary of the characteristics and description of these data for clarity. To ensure consistency in data extraction, two researchers independently registered the studies, cross-verifying all extracted data with the sources.

**Table 2.** Summary of the studies included according to the answers to the review question for each study

Authors, title	Subject área, country	Study objective	Method, data collection instrument, participants	Main results
Rosenthal and Dawson (1991), Wives of Institutionalised Elderly Men. The first Stage of the transition to Quasi-Widowhood.	Medicine, Canada	To consider the experiences of wives whose husbands have entered a long-term care institution	(a) Qualitative, conceptual model of the transition to quasi-widowhood; (b) Interview; (c) 69 women with a spouse in long-term care facilities.	Quasi-widowhood resembles widowhood, especially in life reconstruction, with a spouse. Most women perceive their marriage as stable or declining, with uncertainty about the future. The initial post-admission period poses emotional and physical health risks, characterized by crisis, guilt, loneliness, and vulnerability.
Gladstone (1992), Identifying the living arrangements of Elderly Married Couples in long-term Care Institutions.	Social Sciences, Canada	To study married couples and elderly married individuals in homes for the aged and in nursing homes	(a) Quantitative; (b) questionnaire; (c) 454 administrators for homes for the aged (HFA) and nursing homes (NH).	The distribution of couples differs between homes for the aged (HFA) and nursing homes (NH). HFA has more resident couples, while in NH, most spouses live in the community. Room separation occurs due to cognitive impairment (39%) or personal choice (25%).
Gladstone (1995a), living in long term Care Institutions: A qualitative analysis of feelings.	Social Sciences, Canada	To study the feelings that institutionalised and community-dwelling spouses have in regard to their own or their spouse’s living in a long-term care facility	(a) Qualitative, analysis of data from open-ended questions; (b) Interview; (c) of the 161 respondents, 87 were both living in institutions, and 74 lived in the community but had a husband or wife in an institution.	Marital issues were linked to spouse dependence and staff intrusion, hindering intimacy and conflict management. Both institutionalized and community-dwelling spouses reported relief from household chores and positive feelings about institutionalization. The primary reason for admission was one spouse’s declining health and the inability to provide adequate care.
Gladstone (1995b), the marital perceptions of elderly persons living or having a spouse living in a long-term care institution in Canada.	Social Sciences and Humanities, Canada	To understand the way that older married persons living, or having a spouse living, in a long-term care institution perceive their marriages following relocation.	(a) Qualitative, Grounded Theory; (b) Interview; (c) 161 participants, including 31 married couples, lived with their spouses in institutions	Higher-educated spouses provided more care. Physical and emotional contact, visits, conversations, and activities helped sustain marriages, preserve conjugal roles, and provide purpose. Interviewees affirmed their marital identities within the institution.
Ross et al. (1997), Spousal caregiving following institutionalisation: The experience of elderly wives.	Nursing, USA	To understand nurses’ awareness, through a review of the literature on family caregiving in later life, of the contribution to care made by elderly wives following the institutionalisation of their husbands	a) Quantitative and qualitative. Content analysis by Berg; b) Interview, questionnaire; c) Seventy-eight wives whose spouses were institutionalised.	The primary reasons for institutionalization included falls, incontinence, dysphagia, seizures, memory loss, confusion, and aggression. Visits were motivated by duty, assistance, and monitoring well-being. Care aimed to preserve husbands’ personalities, family ties, and counter institutional standardization. Marital closeness was sustained through visits and the concept of couple continuity.
Lundh et al. (2000), “I don’t have any other choice”: spouses’ experiences of placing a partner in a care home for older people in Sweden.	Nursing, Sweden	To consider the experiences of Swedish spouses who have placed a partner in a care home for older people	(a) Qualitative, grounded theory; (b) Interview, field notes; (c) 14 spouse carers, 11 wives and 3 husbands.	The institutionalization process is understood temporally in four dimensions: Decision-making, transition, adjustment, and reorientation. Caregivers emphasize continuity of care and involvement. Transitions to long-term care are often obstructed by staff. Further research is needed to better understand the institutionalization of the older adults.
Braithwaite (2002), “Married widowhood”: Maintaining a couple’s relationship when one spouse is living in a nursing home.	Public health Sciences, UK	To explore the role additions and deletions for community-based wives whose husbands moved to a nursing home	(a) Qualitative, Content analysis by Kaplan; (b) Interview; (c) 21 wives whose spouses resided in a nursing home.	Couples adapt their identities to maintain long-term relationships, categorized into no couplehood, low couplehood, and high couplehood. Women with stronger marital bonds use “we” instead of “I.” A deeper understanding is needed of how couples preserve their identities and relationships, supported by formal and informal networks.
Sandberg et al. (2001), placing a spouse in a care home: the importance of keeping.	Nursing, Sweden	To explore the experience of spouses who have placed a partner in care.	(a) Qualitative, Grounded theory; (b) Interview, field notes; (c) 14 spouses (11 wives and three husbands) with partners living in care homes.	The emotional instability of the community-dwelling spouse often goes unnoticed by family and professionals. Caregivers promote marital continuity through joint activities. Relationship maintenance includes contact, specialness, and vigilance, with professional care emphasizing the latter. The placement process often overlooks spouses’ emotional responses and efforts to sustain their relationship.
Stadnyk (2006), Community-dwelling spouses of nursing home residents: Activities that sustain identities in times of transition.	Public health Sciences, Canada	To explore the role of activities in the transitions of community-dwelling married persons that are related to placing their spouse in a nursing home	(a) Qualitative; Content analysis according based on literature. Does not specify author or framework used; (b) Interviews; (c) 52 Community-dwelling spouses of nursing home residents.	Joint activities in nursing homes help sustain marital identity for the community-dwelling spouse. The transition marks the end of domestic life, termed “married widowhood.” limited privacy and shared spaces hinder conjugal intimacy. Participants experienced common challenges in adjusting to a spouse’s move to a nursing home.
Martin et al. (2008), an exploration of spousal separation and adaptation to long-term disability: six elderly couples engaged in a horticultural program.	Ocupacional therapy, USA	To explore the impact of separation on couples where one spouse lives in a skilled nursing facility and the other spouse lives alone in the community	(a) Qualitative, phenomenological and hermeneutic methods; (b) Interview, observation; (c) Six spouses, with partners in the community.	Key concepts identified include isolation, routine and activity changes, and sacrifices in the marital relationship. Adaptation involved an internal struggle between preserving the past and adjusting to new circumstances. Separation altered communication and affection. Further research is needed on role changes and identity preservation in relationships.
Eriksson and Sandberg (2008), transitions in men’s caring identities: Experiences from home-based care to nursing home placement.	Nursing, Sweden	To describe, from a gender identity perspective, the experiences of older men involved in the process of caring for a partner at home and the placement into a nursing home	(a) Qualitative, constructivist approach; (b) Interview; (c) Seven men with a spouse in a nursing home.	The practice of caregiving shapes gender identities through love, care, and friendship, influencing male caregiver identity. As wives transition to nursing homes, relationships shift toward friendship, redefining care intimacy. Men’s adaptation highlights gender identity challenges, emphasizing the need for further research on caregiving transitions.
Førsund et al. (2014), the loss of a shared lifetime: a Qualitative study exploring spouses’ experiences of losing *couplehood* with their partner with dementia living in institutional care.	Nursing, Norway	To explore and describe spouses’ experiences of losing couplehood with their dementia-afflicted partner living in institutional care.	(a) Qualitative, Grounded Theory; (b) Interview, field notes; (c) 10 spouses (five men, five women) with partner living in institutional care.	Physical separation from a partner induces loneliness and absence. Spouses compensate through visits and joint activities. Three key experiences emerged: loss of shared daily life, loss of a shared past linked to home and memories, and loss of a shared future, causing internal conflict.
Hennings et al. (2015), Spouse caregivers of people with advanced dementia in nursing homes: A longitudinal narrative study.	Medicine, UK	To explore the caregiving experiences of spouse carers of people with advanced dementia living in nursing homes.	(a) Qualitative, not specified author or framework used (b) Interviews, diary writing; (c) ten spouse caregivers (7 women and 3 men).	The caregiving experience is defined by love, care, and friendship. Transition is crucial for adapting between the nursing home and society. Key caregiver narratives reflect emotional struggles and social loss. Diverse interventions are needed, requiring further research to develop effective support strategies for caregivers.
Kemp et al., (2016), Couples’ Social Careers in Assisted living: Reconciling Individual and Shared Situations.	Medicine, USA	To advance knowledge by gaining an in-depth understanding of married and unmarried couples’ intimate and social relationships in assisted living	(a) Qualitative, Grounded Theory; (b) Interviews, observations, field notes, health and social network data; (c) 29 couples in assisted living (AL).	Marital advantages included companionship, support, and affection, while disadvantages involved caregiver burden and restricted choices. Couples were categorized into various typologies. Later-life intimacy was evident. Support groups were valuable, highlighting the need for research on diverse living arrangements and partnerships.
Olsen et al. (2019), A spouse in the nursing home: The conflicting experience of separation.	Nurse, Norway	To look at love and marriage structures during the lifetime of the agents.	(a) Qualitative, Bourdieu’s theory; (b) Interviews, historical analysis; (c) Five interviews with two women and two men who have spouses in nursing homes	The transition to a nursing home. Has affected spousal privacy, resulting in daily visits characterized by sadness and dissatisfaction. Promoting discussions of life stories and welcoming couples is essential for mitigating feelings of loneliness and rejection.
Soulsby et al. (2022), The Husband: Navigating the relational Challenge of Her Institutionalization or His Widowerhood.	Psychology, sociology and anthropolo-gy, USA and UK	To explore how men’s relational identity as husband is maintained despite challenges, both during and after the marriage ends.	(a) Qualitative, Grounded Theory; (b) Interview; (c) 12 US husbands (who have spouses in long-term care); 12 British widowers.	Marital interactions occur within a structured context, in collaboration with the professional team. The marital relationship evolves through various phases, from affectionate to distant, with husbands seeking fulfillment through visits. Further research is needed to explore how husbands maintain relational identity after placement.
Shaw and Csikai (2024), The preservation of Spousal and partner relationships among nursing home residents	Social Sciences, USA	To explore services and activities offered that emphasise the preservation of spousal and partner relationships among nursing home residents	(a) Mixed-method research; Not specified author or framework used (b) questionnaire, interviews; (c) 34 nursing home social workers	Nursing homes employ practices to preserve marital relationships, such as ensuring privacy, cohabitation requests, private space, and facilitating outings. However, only a small percentage of facilities offer programs focused on relationship preservation, with insufficient private areas for marital interactions.

We analyzed qualitative data through an inductive thematic synthesis following the framework described by Thomas and Harden (2008). We systematically coded the extracted data to identify recurring patterns and concepts relevant to conjugal relationships within different living arrangements (Spouses living in LTCFs with partners in the community, couples co-residing in LTCFs, and key findings shared between both scenarios). We then grouped the codes into overarching themes that reflect the diversity of conjugal experiences in LTC settings. The research team held iterative discussions to reach consensus, enhance interpretative validity, and ensure analytical rigor. We present a detailed synthesis of these findings in Table 3.

**Table 3.** Key themes on living arrangements in LTCFs

Spouses living in LTCFs with partners in the community	Couples co-residing in LTCFs	Studies addressing both scenarios
Spousal care (Braithwaite, 2002; Eriksson & Sandberg, 2008; Hennings et al., 2015; Olsen et al., 2019; Sandberg et al., 2001). A difficult decision (Lundh et al., 2000; Martin et al., 2008; Rosenthal & Dawson, 1991; Ross et al., 1997; Soulsby et al., 2022). Spousal separation (Forsund et al., 2014; Hennings et al., 2015; Lundh et al., 2000; Martin et al., 2008; Olsen et al., 2019; Rosenthal & Dawson, 1991; Soulsby et al., 2022; Stadnyk, 2006). Maintaining the couple’s identity (Braithwaite, 2002; Ross et al., 1997; Sandberg et al., 2001; Stadnyk, 2006). Positive and negative experiences (Eriksson & Sandberg, 2008; Forsund et al., 2014; Hennings et al., 2015; Martin et al., 2008; Olsen et al., 2019; Rosenthal & Dawson, 1991). Intimacy and privacy (Eriksson & Sandberg, 2008; Hennings et al., 2015; Olsen et al., 2019; Stadnyk, 2006). Transition process (Hennings et al., 2015; Lundh et al., 2000; Martin et al., 2008; Olsen et al., 2019; Rosenthal & Dawson, 1991). Policy frameworks (Hennings et al., 2015; Lundh et al., 2000; Olsen et al., 2019; Rosenthal & Dawson, 1991; Ross et al., 1997).	Share the same room (Gladstone, 1992; Kemp et al., 2016; Shaw & Csikai, 2024). Relationship preservation practices (Gladstone, 1992; Shaw & Csikai, 2024). Positive and negative feelings (Kemp et al., 2016). Intimacy and privacy (Kemp et al., 2016; Shaw & Csikai, 2024). Transition process (Kemp et al., 2016). Policy frameworks (Shaw & Csikai, 2024).	Positive and negative feelings (Gladstone, 1995a). A difficult decision (Gladstone, 1995b). Maintaining the couple’s identity (Gladstone, 1995b). Intimacy and privacy (Gladstone, 1995a, 1995b).

## Results

### Characteristics of the Analysed Studies

The sample included 17 articles that we reported in chronological order from 1991 to 2024 (Table 4).

**Table 4.** Descriptive Summary of Results With Frequency and Percentages

	Papers (*N*)	%
Publication year		
1990–1994	2	11.8
1995–999	3	17.6
2000–2004	3	17.6
2005–2009	3	17.6
2010–2014	1	5.9
2015–2019	3	17.6
2020–2024	2	11.8
Study Country		
Canada	5	29.4
United States of America	4	23.5
Sweden	3	17.6
United Kingdom	2	11.8
Norway	2	11.8
Partnership between United States of America and United Kingdom	1	5.9
Subject area		
Nursing	6	35.3
Social Sciences	5	29.4
Health Sciences	3	17.6
Medicine	2	5.9
Occupational therapy	1	11.8
Study design		
Qualitative	14	82.3
Mixed methods	2	11.8
Quantitative	1	5.9
*Implementation theory or framework used		
Grounded theory	7	41.2
Model of the transition	1	5.9
Content analysis according to Berg	1	5.9
Phenomenology	1	5.9
Bourdieu’s theory	1	5.9
The narrative study	1	5.9
Mixed method	2	11.8
Quantitative typology	1	5.9
Not specified author or framework used	2	11.8
Data collection method^a^		
Interview	14	82.4
Questionnaire	2	11.8
Field notes	3	17.6
Observation	2	11.8
Historical analysis	1	5.9
Diary writing	1	5.9
Health and social network data	1	5.9
More than one	2	11.8
Living arrangements		
Spouses living in LTCFs with partners in the community	12	70.6
Couples co-residing in LTCFs	3	17.6
Studies addressing both scenarios	2	11.8

Notes: *N* = 17.

^a^Some studies used more than one framework or data collection instrument, and the sum of the categories is greater than the total number of studies.

Analyzing the geographical origin of the articles based on the country where the study was conducted, Canada produced the highest number of publications (Table 4). The studies primarily focused on nursing knowledge (*n* = 6), social sciences (*n* = 5), health sciences (*n* = 3), medicine (*n* = 2), and occupational therapy (*n* = 1).

All studies explored feelings and experiences related to marital relationships in residential facilities for older adults. Methodologically, the majority of the studies (70.6%, *n* = 14) employed qualitative approaches, two (11.8%) used mixed methods, and one (5.9%) applied a quantitative approach. Grounded Theory was the predominant theoretical framework (*n* = 7), followed by Content Analysis (*n* = 2), Phenomenology (*n* = 1), Bourdieu’s Theory (*n* = 1), and Narrative Analysis (*n* = 1). Two studies did not specify the theoretical framework used. The two quantitative studies applied descriptive methodologies.

Researchers primarily collected data through semi-structured interviews (*n* = 14). Additional methods included questionnaires (*n* = 1), mixed interviews and questionnaires (*n* = 2), field notes (*n* = 3), direct observation (*n* = 2), document analysis (*n* = 1), diary writing (*n* = 1), and health/social network data (*n* = 1).

Regarding sample characteristics (Table 4), most studies (70.6%, *n* = 12) focused on dyads where one spouse lived in a LTCF and the other remained in the community. A smaller number examined co-residing couples in long-term care facilities (17.6%, *n* = 3) or included both arrangements (11.8%, *n* = 2).

A. Spouses living in LTCF with partners in the community
• A difficult decision

Several studies described the difficult decision to move one spouse into an LTCF. Soulsby et al. (2022) describe this experience as becoming “a married widow” or a “widow.” Sandberg et al. (2001) reported that such a decision typically prioritizes the well-being of the spouse remaining at home. Other studies (Gladstone, 1995b; Martin et al., 2008; Rosenthal & Dawson, 1991; Ross et al., 1997; Sandberg et al., 2001) linked this decision to reduced capacity for managing daily activities or health deterioration.

• Spousal separation

Older adult couples often described separation as involuntary. Separating from a spouse and adapting to “two worlds” is defined as a complex process (Hennings et al., 2015; Rosenthal & Dawson, 1991). Martin et al. (2008) linked separation to changes in communication and expressions of affection due to the cognitive impairment of the spouse receiving care. Several studies reported a progressive withdrawal from the external environment, particularly from the broader community (Lundh et al., 2000; Martin et al., 2008; Olsen et al., 2019; Soulsby et al., 2022). Additionally, Førsund et al. (2014) confirmed the physical absence of the partner and the experience of separation. Martin et al. (2008) and Stadnyk (2006) described spousal separation as a devastating experience.

B. Couples co-residing in LTCFs
• Sharing the same room

All studies on couples co-residing in LTCFs indicate that sharing a room in later life contributes to the preservation of intimacy (Gladstone, 1992; Kemp et al., 2016; Shaw & Csikai, 2024). However, Gladstone (1992) found that 38% of couples sleep separately, primarily due to cognitive impairment. Kemp et al. (2016) reported that long-term relationships provided companionship, affection, and mutual care. However, cohabitation also posed challenges. The caregiving burden limited individual choices and reduced autonomy, as the needs of the care recipient imposed significant restrictions. Shaw and Csikai (2024) confirmed that sharing the same room is essential for sustaining intimacy.

• Relationship preservation practices

Shaw and Csikai (2024) documented the lack of private spaces for couples’ intimacy and the difficulties social workers face in accommodating this need. Supportive practices included ensuring privacy upon request, accommodating room-sharing, granting access to private areas, and supporting outings. However, only a few LTCFs offered specific programs to maintain marital relationships. Gladstone (1995a) found that marital preservation was hindered by partner dependence, interference from other residents, and professional involvement in the conjugal dynamic. These factors compromised marital intimacy and complicated the management of spousal conflict.

C. Key findings shared by both scenarios
• Spousal care

When one spouse moves into residential care, the experiences of older couples are associated with spousal care (Eriksson & Sandberg, 2008; Hennings et al., 2015; Sandberg et al., 2001). Caring for the partner was described as enjoyable, and spouses expressed satisfaction associated with these caregiving activities (Ross et al., 1997). Providing care, adapting to facility routines, and making daily visits gave the caregiving spouse a renewed sense of purpose in life (Hennings et al., 2015; Olsen et al., 2019; Soulsby et al., 2022).

• Maintaining the couple’s identity

Maintaining the couple’s identity emerged in the scientific literature as a recurring theme. Ross et al. (1997) introduced this concept, further reinforced by Sandberg et al. (2001). Spouses affirmed their identities as married individuals, valuing activities that preserved emotional and relational bonds (Braithwaite, 2002; Gladstone, 1995b; Ross et al., 1997; Stadnyk, 2006). Frequent presence and involvement in the partner’s life sustained relational identity (Lundh et al., 2000). The couple identity, shaped by marriage and a shared sense of “we”, faced challenges in long-term relationships (Braithwaite, 2002; Olsen et al., 2019; Rosenthal & Dawson, 1991; Soulsby et al., 2022). Emotional and physical closeness supported this identity (Gladstone, 1995b). Hennings et al. (2015) and Kemp et al. (2016) called for support programs and resource centers to foster joint activities and strengthen marital bonds.

• Positive and negative experiences

Several studies in our sample reported a range of emotional experiences during the transition to residential care. Spouses frequently expressed loneliness and isolation (Førsund et al., 2014; Martin et al., 2008; Olsen et al., 2019; Rosenthal & Dawson, 1991), which some facilities addressed through joint activities (Førsund et al., 2014). Gladstone (1995a, 1995b) identified mixed emotions post-relocation, including relief, freedom, and acceptance, alongside reports of distant or tense marital dynamics. Despite challenges, couples often expressed enduring love, affection, and caregiving (Eriksson & Sandberg, 2008; Hennings et al., 2015; Kemp et al., 2016). Friendship remains an essential element (Førsund et al., 2014), and Soulsby et al. (2022) emphasized love as a lasting emotional bond in later-life partnerships.

• Intimacy and privacy

We found that in many studies, the spaces of residential care are not conducive to the couple’s conjugal intimacy/privacy. These two concepts are shared by both scenarios in our sample, as factors influencing the marital relationship (Eriksson & Sandberg, 2008; Gladstone, 1995a; Hennings et al., 2015; Kemp et al., 2016; Shaw & Csikai, 2024; Stadnyk, 2006). Professionals’ presence often deters the couple’s intimacy (Gladstone, 1995b). Olsen et al. (2019) observed that the privacy of the home was transferred to the public domain of the residential facility. Thus, the spouse who remains in the community is regarded as a visitor in the environment where their partner now resides.

• Transition process

The theme of transition was identified as crucial to both intimate experiences and the process of adaptation within the institutional environment (Hennings et al., 2015; Rosenthal & Dawson, 1991). Hennings et al. (2015) emphasized its significance in the context of intimacy. Martin et al. (2008) described how entering residential care settings alters marital relationships and forces changes in couples’ roles. They identified adaptive strategies to manage institutional routines, such as staying calm and flexible to remain attentive to one’s partner and to adjust to the new environment. Kemp et al. (2016) and Olsen et al. (2019) detailed a gradual transition process, beginning with in-home care, followed by temporary stays in care facilities, and eventually leading to permanent residence. This staged transition supported adjustment to the institutional setting.

• Policy frameworks

Lundh et al. (2000) reported that healthcare professionals rarely supported or facilitated couples’ transitions to LTCFs. This difficulty is reported by spouses still residing in the community, who experience it as two different worlds (Hennings et al., 2015; Rosenthal & Dawson, 1991). Ross et al. (1997) identified the importance of moving away from standardized toward individualized practices. These standardized approaches and regulations limit the participation of spouses (Olsen et al., 2019). Similarly, Shaw and Csikai (2024) emphasized the need to develop policies that ensure the preservation of privacy and intimacy in long-term care settings.

## Discussion

This review underscores a growing yet underexplored area in gerontological research: conjugal relationships within LTCFs. Most existing studies examine situations where only one spouse is institutionalized (e.g., Braithwaite, 2002; Soulsby et al., 2022), while far fewer address the experiences of co-residing couples.

Living arrangements significantly shape marital dynamics in distinct ways. For spouses separated by admission, key themes include “difficult decision” and “spousal separation”, with substantial emotional consequences (Soulsby et al., 2022). These couples often experience role ambiguity and a loss of shared conjugal identity (Torgé, 2020; Stefansdottir et al., 2024). In contrast, co-residing couples emphasized themes such as “sharing the same room” and “relationship preservation”, though they still face limitations in intimacy and privacy (Shaw & Csikai, 2024).

Several themes cut across both contexts: spousal care, efforts to maintain couple identity, mixed feelings, and institutional constraints. Spousal care emerged as a central theme, cutting across diverse living arrangements. When partners experience multiple limitations in activities of daily living (ADL), caregiving demands intensify both physically and emotionally (Teles et al., 2024). In this regard, Stefansdottir et al. (2024) demonstrate that the transition to LTCFs significantly affects marital roles, with caregivers’ health and functional capacity playing a crucial role in this process (Tate, Bailey, Deschenes, Grabusic, & Cummings, 2023). Balancing caregiving demands with personal needs remains a persistent challenge (Torgé, 2020). Within institutional settings, emotions such as guilt, grief, and anxiety are common (Hähnel et al., 2023), often stemming from role ambiguity and loss of personal identity (Torgé, 2020). These are further exacerbated when the spouse has cognitive impairments (Toot et al., 2017).

Although caregiving responsibilities remain present, some spouses report improvements in emotional well-being following their partner’s admission to a LTCF (Liu et al., 2019). Improved access to medical support (Lin et al., 2024) and increased opportunities for social engagement for both partners may contribute to a greater sense of stability (Kim et al., 2024).

Although awareness of conjugal well-being is growing, current supportive practices remain inadequate. Shared rooms, flexible visitation, and designated couple activities are rare exceptions rather than the norm (Hennings et al., 2015; Kemp et al., 2016). Institutional routines often disrupt intimacy, with staff interactions unintentionally eroding emotional connection (Gladstone, 1995b; Olsen et al., 2019). Even couples who live together may feel reduced to the role of patients rather than relational partners.

It is important to note that spouses residing outside the facility are frequently treated as visitors instead of caregivers. This restricts their involvement in care and undermines conjugal bonds (McArthur et al., 2021; Ross et al., 1997). Structured interventions, such as caregiver participation, joint activities, and psychosocial support, can mitigate this disconnection and foster resilience (Kemp et al., 2016).

From a policy standpoint, Shaw and Csikai (2024) stress the urgent need for relationship-centered care models. Professional interference remains a significant barrier to intimacy within LTC settings (Gladstone, 1995b; Olsen et al., 2019). In line with these findings, healthcare professionals must receive training to recognize the importance of conjugal relationships for the well-being of residents in LTCFs and to avoid actions that may hinder their expression (Ahlström et al., 2021; McAuliffe et al., 2023; Torgé, 2020).

Training programs should prioritize relational continuity, intimacy, and the psychosocial challenges of institutional adaptation. Care practices must support rather than disrupt existing marital bonds. Recognizing couples as relational units enables care teams to tailor interventions and foster a care culture that promotes both individual and shared well-being (Gurung & Chaudhury, 2025; Larkey, 2021).

## Limitations

Although the review’s rigor, several methodological limitations remain. The predominance of qualitative studies (70.6%) limits generalizability. The scarcity of recent, quantitative, and longitudinal studies restricts our understanding of how marital relationships evolve within LTCFs. Geographic and care facility variability further challenge findings, as national healthcare systems and LTCF policies vary widely, affecting marital adaptation. Linguistic bias led to the inclusion of mainly English-language studies, potentially excluding relevant research from non-Western regions where family caregiving traditions differ (Fabian de Leva et al., 2025; Ikeorji & Warria, 2024; Oesterdiekhoff, 2024; Rostgaard et al., 2022; Shrestha et al., 2024). Additionally, heterogeneity in assessment tools and conceptual frameworks further complicates cross-study synthesis and comparability.

## Future Research Directions

Despite the growing recognition of marital well-being in aging research, few studies explore how institutional policies and national regulations influence conjugal relationships. Future research should extend comparative analyses to assess the impact of different LTC systems on marital bonds, privacy rights, and spousal roles. There is also a need for cross-cultural studies examining LTCF policies outside of Western contexts, particularly in regions where family-based caregiving is predominant.

To advance research on conjugal relationships in LTCFs, future studies should prioritize quantitative and longitudinal approaches to track relational dynamics over time. Integrating cross-cultural comparisons will provide insights into how healthcare policies, residential care practices, and cultural norms influence marital well-being in LTC settings. Expanding research beyond English-language literature will ensure more inclusive and global findings.

Addressing these methodological gaps can contribute to the development of evidence-based interventions that support conjugal well-being in LTCFs. Ultimately, recognizing relational continuity as a core component of aging policies will be crucial for enhancing quality of life, marital well-being, and autonomy among older couples in these settings.

## Conclusions

This review highlights the vital importance of preserving conjugal identity among older couples residing in LTCFs. Emotional bonds, intimacy, and privacy are fundamental to partners’ well-being and are deeply influenced by institutional environments and living arrangements, including opportunities for cohabitation. Conjugal relationships constitute an essential dimension of quality of life in LTCFs; however, current policies and practices often fail to support relational continuity. Addressing this gap requires the implementation of relationship-centered care models that provide private spaces, dedicated resources, and structured interventions. Furthermore, comprehensive training of healthcare professionals is imperative to enhance awareness and sensitivity to conjugal dynamics, thereby ensuring care that respects and reinforces marital relationships within institutional contexts.

## Supplemental Material

Supplemental Material - Experience of Conjugality Among Older People in Long-Term Care Facilities: A Scoping Review Supplemental Material for Experience of Conjugality Among Older People in Long-Term Care Facilities: A Scoping Review by Florbela Bia, Zaida Charepe, and Cristina Marques-Vieira in Journal of Applied Gerontology

## Data Availability

All data supporting the findings of this study were derived from publicly available sources. The extracted and synthesized data are available upon reasonable request from the corresponding author.*
