# Supplementary material for: Experience of Conjugality Among Older People in Long-Term Care Facilities: A Scoping Review
Source: J Appl Gerontol. 2025 Aug 26;45(7):1301–15. doi: 10.1177/07334648251369264 (PMC13260767; doi:10.1177/07334648251369264)
Supplement: Supplemental Material - Experience of Conjugality Among Older People in Long-Term Care Facilities: A Scoping Review [file sj-pdf-1-jag-10.1177_07334648251369264.pdf]

Supplementary File 1. Strategy, research limiters and results applied to each database.

Database: CINAHL Complete (via EBSCO)<br>Results: 220<br>Research strategy (December 2023))
(SU ("late age" OR "geriatric people" OR elderly OR "older adults" OR "late-life")) OR MH ((Aged OR aging))) AND ((SU (Conjugality OR "marital perceptions" OR "marital satisfaction" OR "marital relationship*" OR "marriage pattern*" OR Couples OR "conjugal intimate" OR "Marital perceptions") OR MH ((Marriage OR "marital status"))) AND ((SU ("institutions of long permanence" OR "old age homes" OR "Nursing Home Patients" OR "institutionaliz*")) OR MH (("Long- Term Care" OR "housing for the elderly" OR "senior centers" OR "nursing homes" OR "Nursing Home Patients"))
Database: MedicLatina (via EBSCO)<br>Results: 5<br>Research strategy (December 2023)
((TI ("late age" OR "geriatric people" OR elderly OR "older adults" OR "late-life" OR Aged OR aging) OR AB ("late age" OR "geriatric people" OR elderly OR "older adults" OR "late-life" OR Aged OR aging) OR SU ("late age" OR "geriatric people" OR elderly OR "older adults" OR "late-life" OR Aged OR aging)) AND (TI (Conjugality OR "marital perceptions" OR "marital satisfaction" OR "marital relationship*" OR "marriage pattern*" OR Couples OR "conjugal intimate" OR "Marital perceptions" OR Marriage OR "marital status") OR AB (Conjugality OR "marital perceptions" OR "marital satisfaction" OR "marital relationship*" OR "marriage pattern*" OR Couples OR "conjugal intimate" OR "Marital perceptions" OR Marriage OR "marital status") OR (Conjugality OR "marital perceptions" OR "marital satisfaction" OR "marital relationship*" OR "marriage pattern*" OR Couples OR "conjugal intimate" OR "Marital perceptions" OR Marriage OR "marital status")) AND (TI ("institutions of long permanence" OR "old age homes" OR institutionaliz* OR "Long Term Care" OR "housing for the elderly" OR "senior center*" OR "nursing home*") OR AB ("institutions of long permanence" OR "old age homes" OR institutionaliz* OR "Long Term Care" OR "housing for the elderly" OR "senior center*" OR "nursing home*") OR SU ("institutions of long permanence" OR "old age homes" OR institutionaliz* OR "Long Term Care" OR "housing for the elderly" OR "senior center*" OR "nursing home*")))
Database: MEDLINE Complete (via EBSCO)<br>Results: 96<br>Research strategy (December 2023)
((SU ("late age" OR "geriatric people" OR elderly OR "older adults" OR "late-life")) OR MH ((Aged OR aging)) AND ((SU (Conjugality OR "marital perceptions" OR "marital satisfaction" OR "marital relationship*" OR "marriage pattern*" OR Couples

OR "conjugal intimate" OR "Marital perceptions") OR MH ((Marriage OR "marital status"))) AND ((SU ("institutions of long permanence" OR "old age homes" OR "Nursing Home Patients" OR "institutionaliz*")) OR MH (("Long-Term Care" OR "Homes for the Aged" OR "housing for the elderly" OR "senior centers" OR "nursing homes")))
Database Psychology and Behavioral Sciences Collection (via EBSCO)<br>Results: 2<br>Research strategy (December 2023)
(((SU ("late age" OR "geriatric people" OR elderly" OR "older adults" OR "late-life" OR Aged OR aging)))) AND ((SU Conjugality OR "marital perceptions" OR "marital satisfaction" OR "marital relationship*" OR "marriage pattern*" OR Couples OR "conjugal intimate" OR "Marital perceptions" OR Marriage OR "marital status"))) AND ((SU ("institutions of long permanence" OR "old age homes" OR "Nursing Home Patients" OR "institutionaliz*" OR "Long Term Care" OR "housing for the elderly" OR "senior centers" OR "nursing homes" OR "Nursing Home Patients")))
Database: PsycArticles (via EBSCO)<br>Results: 3<br>Research strategy (December 2023)
(((SU ("late age" OR "geriatric people" OR elderly" OR "older adults" OR "late-life" OR Aged OR aging)))) AND ((SU Conjugality OR "marital perceptions" OR "marital satisfaction" OR "marital relationship*" OR "marriage pattern*" OR Couples OR "conjugal intimate" OR "Marital perceptions" OR Marriage OR "marital status"))) AND ((SU ("institutions of long permanence" OR "old age homes" OR "Nursing Home Patients" OR "institutionaliz*" OR "Long Term Care" OR "housing for the elderly" OR "senior centers" OR "nursing homes" OR "Nursing Home Patients")))
Database: PubMed (via EBSCO)<br>Results: 203<br>Research strategy (December 2023)
(("late age"[Title/Abstract] OR "geriatric people"[Title/Abstract] OR "elderly"[Title/Abstract] OR "older adults"[Title/Abstract] OR "late-life"[Title/Abstract] OR "aged"[MeSH Terms] OR "ageing"[MeSH Terms]) AND ("conjugality"[Title/Abstract] OR "Marital perceptions"[Title/Abstract] OR "marital satisfaction"[Title/Abstract] OR "marital relationship*"[Title/Abstract] OR "marriage pattern"[Title/Abstract] OR "Couples"[Title/Abstract] OR "marriage"[MeSH Terms] OR "marital status"[MeSH Terms])) AND ("old age homes"[Title/Abstract] OR "Nursing Home patients"[Title/Abstract] OR "institutionaliz*"[Title/Abstract] OR "long term care"[MeSH Terms] OR "housing for the elderly"[MeSH Terms] OR "senior centers"[MeSH Terms] OR "nursing homes"[MeSH Terms])
